# No Biological Evidence of XMRV in Blood or Prostatic Fluid from Prostate Cancer Patients

**DOI:** 10.1371/journal.pone.0036073

**Published:** 2012-05-16

**Authors:** Ramon Mendoza, Robert H. Silverman, Eric A. Klein, A. Dusty Miller

**Affiliations:** 1 Human Biology Division, Fred Hutchinson Cancer Research Center, Seattle, Washington, United States of America; 2 Department of Cancer Biology, Lerner Research Institute, Cleveland Clinic, Cleveland, Ohio, United States of America; 3 Glickman Urological and Kidney Institute, Cleveland Clinic, Cleveland, Ohio, United States of America; 4 Department of Pathology, University of Washington, Seattle, Washington, United States of America; Burnet Institute, Australia

## Abstract

**Background:**

XMRV (xenotropic murine leukemia virus-related virus) was initially discovered in association with prostate cancer and later with chronic fatigue syndrome (CFS). Its association with CFS is now largely discredited, and current results support a laboratory origin for XMRV with no reproducible evidence for infection of humans. However, some results indicating the presence of XMRV in prostate cancer are difficult to attribute to sample contamination. Here we have sought biological evidence that might confirm the presence of XMRV in prostate cancer samples previously having tested positive.

**Methods and Results:**

We have tested for infectious XMRV and neutralizing antibodies against XMRV in blood plasma from 29 subjects with prostate cancer, and for infectious XMRV in prostate secretions from another five prostate cancer subjects. Nine of these subjects had previously tested positive for XMRV by PCR or by virus assay. We did not detect XMRV or related retroviruses in any sample, and the neutralizing activities of the plasma samples were all very low, a result inconsistent with XMRV infection of the plasma donors.

**Conclusions:**

We find no evidence for XMRV infection of any human subject tested, either by assay for infectious virus or for neutralizing antibodies. Our results are consistent with the majority of published studies on XMRV, which find that XMRV is not present in humans. The observed low to undetectable XMRV neutralization by human plasma indicates a lack of innate restriction of XMRV replication by soluble factors in human blood.

## Introduction

The retrovirus XMRV (xenotropic murine leukemia virus-related virus) was initially discovered in human prostate cancer samples [1] and was later found in the blood of a high percentage of patients diagnosed with chronic fatigue syndrome (CFS) [2], raising concern that XMRV was a new human pathogen. However, the majority of subsequent studies have been unable to detect XMRV in humans with or without prostate cancer [3] or CFS [4]. In addition, the XMRV isolates from the early studies were all nearly identical to a virus produced by a commonly used prostate cancer cell line, 22Rv1 [5]–[7]. Perhaps XMRV was present in the prostate cancer from which the 22Rv1 cells were derived, but the lack of XMRV sequence diversity was puzzling given the high mutation rate of retroviruses. Recently, the XMRV present in 22Rv1 cells was shown to have arisen during passage of the 22Rv1 prostate cancer cells and their ancestors in nude mice, by a rare recombination event between two endogenous mouse retroviruses, and was not detected in early xenografts of the prostate tumor [8]. The expected rarity of this event and the lack of sequence diversity in the “human” XMRV isolates [7], [9] suggest that the human samples were contaminated with the 22Rv1 XMRV or plasmid clones of XMRV.

Currently, a role for XMRV in CFS is largely disproven, and the original paper that found this association has been retracted [10]. In particular, a large collaborative study found that two of the laboratory groups involved in the original research could not reliably detect XMRV in patient samples, and that labs that could reliably detect XMRV did not detect XMRV in patients with CFS or in normal controls [11]. In the case of the association of XMRV with prostate cancer, it is still unclear whether some of the original prostate cancer samples might have contained patient-derived XMRV or other related retroviruses.

Here we have analyzed blood plasma and expressed prostatic secretions (EPS) from prostate cancer patients, some of whom previously tested positive for XMRV [1], [12]–[15], for the presence of XMRV and related retroviruses by using an assay for infectious retroviruses. In addition, we tested blood plasma for neutralizing antibodies against XMRV that might limit our ability to detect XMRV in plasma, and would indicate an immune response against XMRV in the plasma donor. We find no evidence for XMRV or related retroviruses, or a neutralizing antibody response against XMRV, in any of the patient samples.

## Results

### XMRV Detection Methods

To detect infectious XMRV and related retroviruses in patient plasma and EPS samples, we used S^+^L^−^ and marker rescue assays that have been shown to effectively detect XMRV [5]. The S^+^L^−^ assay we used measures the ability of a retrovirus to infect and cause spread of the Moloney murine sarcoma virus present in PG-4 cat cells [16], leading to production of transformed foci in the cell layer. The marker rescue assay was performed using *Mus dunni* tail fibroblasts (dunni cells) transduced with a retroviral vector (LAPSN) that produces human placental alkaline phosphatase (AP). The dunni/LAPSN cells were exposed to test samples, were passaged for a month to allow virus spread, and were assayed for production of the LAPSN vector on naive dunni cells. Dunni cells were chosen for this assay because of their susceptibility to a wide range of murine leukemia viruses [17], including XMRV, other xenotropic retroviruses, and polytropic retroviruses of the type previously detected in humans [1], [2], [18]. To detect neutralizing antibodies present in patient plasma samples, we used the S^+^L^−^ assay to quantitate replication-competent XMRV after incubation with the samples, in comparison to XMRV incubated with culture medium as a control. In some experiments, we measured the ability of plasma to neutralize the LAPSN vector packaged in XMRV virions (XMRV-pseudotype LAPSN vector) as a surrogate for direct measurement of XMRV neutralization.

To determine the kinetics of virus spread and the sensitivity of the marker rescue assay, the assay was conducted by exposing dunni/LAPSN cells to 50, 25, 10, 5, 1, or 0 focus-forming units (FFU) of XMRV, as determined by S^+^L^−^ assay. The cells were then assayed weekly for LAPSN production during passage of the cells for a month. LAPSN production was detected at one week following infection with 50 FFU of XMRV, at 2 weeks following infection with 10 and 5 FFU, and at 4 weeks for 1 of 2 plates infected with 1 FFU of XMRV. These results show that the marker rescue assay is approximately as sensitive as the S^+^L^−^ assay for detection of XMRV. However, this marker rescue assay may be more sensitive than the S^+^L^−^ assay for some retroviruses because of the known sensitivity of dunni cells to a wide range of murine retroviruses, while fewer types of murine retroviruses can infect the cat cells used in the S^+^L^−^ assay.

### No Evidence for XMRV Infection of Prostate Cancer Patients

We first tested whether blood plasma from a set of ten prostate cancer patients, three of whom previously tested positive for XMRV by RT-PCR, contained replication-competent XMRV and/or neutralizing antibodies against XMRV (Table 1). We did not detect XMRV or related retroviruses in any sample by S^+^L^−^ assay. To detect neutralizing antibodies, plasma samples were incubated at 1∶10 and 1∶100 dilutions with XMRV-pseudotype LAPSN vector for 30 min at room temperature, and the remaining LAPSN virus was measured by AP^+^ focus assay. Only one of the ten plasma samples showed weak neutralizing activity (neutralizing titer of 10). This neutralizing activity was eliminated by heat inactivation of the plasma, which inactivates complement, showing that no antibodies were present that could directly block virus infection.

**Table 1:** Plasma samples from ten patients with prostate cancer do not contain infectious XMRV or related retroviruses and can neutralize XMRV only partially if at all.

			AP^+^ foci for indicated plasma dilution(HI = heat inactivated plasma)‡			
Patient*	RNase L genotype†	S^+^L^−^ FFU in 30 µl plasma	1∶10	1∶100	HI 1∶10	Neutralizing titer (no HI)
VP124	GA	0	85	161	210	10
**VP234**	AA	0	365	297	266	<10
VP538	AA	0	368	340	224	<10
VP627	GG	0	292	256	273	<10
VP630	GG	0	320	320	257	<10
VP653	GG	0	296	268	222	<10
**VP663**	AA	0	380	286	280	<10
VP673	GA	0	366	258	244	<10
VP683	AA	0	320	250	280	<10
**VP693**	GG	0	364	262	304	<10
No plasma			233	233	188	

* Patient identifiers shown in bold indicate patients who had previously tested positive for XMRV. See Discussion for details.

† Nucleotides at position 1385 of the RNase L coding regions of both patient alleles are shown. A G1385A transition at position 1385 results in a glutamine instead of arginine at amino acid position 462 (R462Q) of RNase L, which has been associated with higher XMRV infection rates in homozygous R462Q patients in some studies [1].

‡ The virus neutralization assay was performed by incubating XMRV-pseudotype LAPSN vector (harvested from human cells infected with XMRV and the LAPSN vector) with plasma samples at the indicated dilutions, or with phosphate-buffered saline as a no plasma control, for 30 min at room temperature. The remaining LAPSN virus was measured by infection of HTX human fibrosarcoma cells and staining for foci of AP^+^ cells two days later. Plasma heat inactivation was performed at 56°C for 30 min. All dilutions were performed using phosphate-buffered saline.

**Figure 1:**
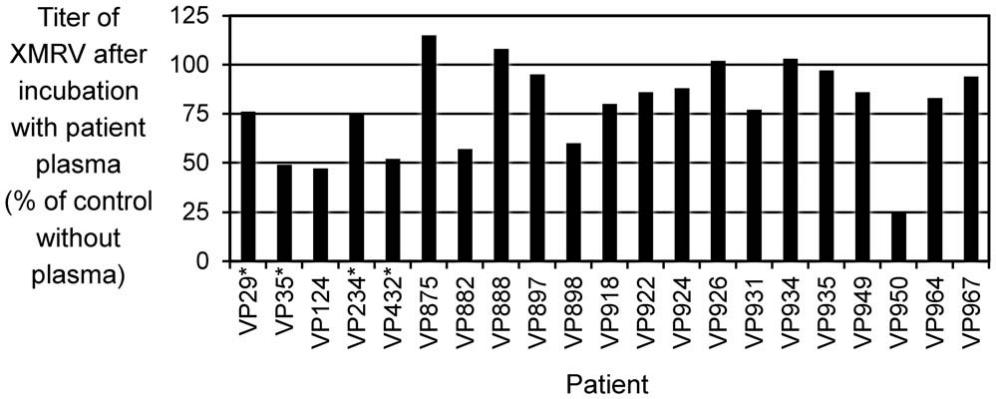
XMRV neutralization by prostate cancer patient plasma. Patient numbers are listed at bottom with asterisks indicating those that had previously tested positive for XMRV (see Discussion for details). The XMRV titer was determined after incubation of a small amount of XMRV with undiluted plasma, or with culture medium as a control, as described in Materials and Methods. Results are shown as the ratio of the XMRV titer after incubation with plasma to that after incubation with culture medium, expressed as a percentage. Note that some values exceed 100%, indicating enhancement of XMRV infection by these plasma samples.

Because of the low to undetectable level of neutralizing antibodies in the first set of 10 patient samples, we conducted additional neutralization assays using undiluted plasma. In addition, we measured neutralization of XMRV virus as opposed to the XMRV-pseudotype LAPSN vector. We did not detect replication-competent XMRV or related retroviruses, by S^+^L^−^ or marker rescue assays, in plasma from any of the 21 patients tested, including four who previously tested positive for XMRV by PCR (see Table 2 for patient and sample details). Furthermore, we found little to no XMRV-neutralizing activity in the undiluted plasma samples, even without heat treatment to inactivate complement (Fig. 1). Sample VP950 showed the highest neutralizing activity (75% neutralization), but heat inactivation of the sample before testing, or diluting the sample 10-fold before testing, abolished the neutralizing activity (data not shown), indicating that this activity is very weak and likely is dependent on complement. The fact that infectious XMRV can persist following incubation with the undiluted plasma samples indicates that infectious XMRV could persist in the blood of these prostate cancer patients, and would be detectable in our assays for replication-competent virus. The apparent lack of a humoral immune response against XMRV suggests that these patients are not infected by XMRV, consistent with our inability to detect virus in these plasma samples.

**Table 2:** Characteristics of plasma and EPS samples tested for infectious XMRV and related retroviruses.

Patient*	RNase L genotype†	Sample	Times frozen	Amount tested by S+L− assay (µl)	Amount tested by marker rescue assay (µl)
**VP29**	AA	Plasma	1	100	100
**VP35**	AA	Plasma	1	100	100
VP124	GA	Plasma	1	100	100
**VP234**	AA	Plasma	1	100	100
		Plasma	2	80	80
**VP432**	AA	Plasma	2	100	100
**VP830**	GA	EPS	2	30	30
**VP844**	GG	EPS	2	50	50
		EPS	3	12	12
VP847	AA	EPS	2	30	30
		EPS	3	20	20
VP875	AA	EPS	2	50	50
		EPS	3	10	10
		Plasma	1	50	50
**VP881**	GA	EPS	2	30	30
VP882	GA	Plasma	1	50	50
VP888	AA	Plasma	1	50	50
VP897	AA	Plasma	1	50	50
VP898	AA	Plasma	1	50	50
VP918	AA	Plasma	1	50	50
VP922	AA	Plasma	1	50	50
VP924	GG	Plasma	1	50	50
VP926	AA	Plasma	1	100	100
VP931	AA	Plasma	1	100	100
VP934	AA	Plasma	1	100	100
VP935	AA	Plasma	1	100	100
VP949	AA	Plasma	1	100	100
VP950	AA	Plasma	1	100	100
VP964	GG	Plasma	1	100	100
VP967	AA	Plasma	1	100	100

* Patient identifiers shown in bold indicate patients who had previously tested positive for XMRV. See Discussion for details.

† Nucleotides at position 1385 of the RNase L coding regions of both patient alleles are shown. See Table 1 footnotes for additional details.

We next tested EPS fluids obtained from excised prostate glands [13] for the presence of XMRV. To test for possible effects of EPS on XMRV infectivity, we added a small amount of XMRV to undiluted EPS from a normal prostate, or to culture medium as a control, and measured the titer of these mixtures by using the S^+^L^−^ assay. Duplicate samples for each mixture gave identical results (XMRV titer of 5×10^6^ FFU/ml), showing that XMRV can survive and be detected in EPS fluid. However, no replication-competent virus was detected in any of 5 EPS samples from prostate cancer patients by S^+^L^−^ or marker rescue assays (see Table 2 for sample identifiers and amounts tested). Three of these patients had previously tested positive for XMRV in urine (Table 2) [15].

### Activation of *M. dunni* Endogenous Retrovirus in Some Marker Rescue Assays

We did experience some false positive results with the marker rescue assay. In a few cases we detected LAPSN production following exposure of dunni/LAPSN cells to plasma, but viral interference analysis showed that LAPSN transduction was completely blocked in dunni cells expressing the *M. dunni* endogenous retrovirus (MDEV) [19], [20], but was unaffected in dunni cells expressing XMRV (data not shown). MDEV and XMRV use different receptors for cell entry, which are blocked by infection with the cognate retrovirus but are unaffected by infection with the alternate virus [17]. To confirm that the positive results were indeed artifactual, we performed a marker rescue assay using DU145/LAPSN cells, and found that all of the apparent false-positive patient samples were indeed negative for replication-competent XMRV (data not shown). Previously, MDEV production from *M. dunni* cells was observed following treatment of the cells with 5-iodo-2′-deoxyuridine or hydrocortisone [19], and it appears that substances in the patient samples have a similar ability to activate the normally silent MDEV locus in dunni cells.

## Discussion

Of the 29 plasma and 5 EPS samples from prostate cancer patients that we tested, none had a detectable level of replication-competent XMRV or related retroviruses. Included were two plasma samples (VP29 and VP35) from patients who tested XMRV positive by viral detection DNA microarray (Virochip) analysis in the original study [1], one plasma sample (VP234) from a patient who tested XMRV positive by RT-PCR of prostate tissue RNA [12], one plasma sample (VP693) from a patient who tested XMRV positive by RT-PCR of RNA isolated from EPS [13], and one plasma sample (VP432) from a patient who tested positive for infectious XMRV in plasma [14]. In particular, note that prostate cancer tissue from patient VP35 was the presumed source of the first full-length clone of XMRV [1]. Also included in our analysis were three EPS samples (VP830, VP844 and VP881) and one plasma sample (VP663) from patients who tested XMRV positive by RT-PCR of RNA isolated from urine [15]. The S^+^L^−^ and dunni cell-based marker rescue assays that we used to detect virus are both capable of detecting xenotropic and polytropic retroviruses [5], [17] of the types previously reported in prostate cancer and CFS patients [1], [2], [18], as well as amphotropic murine leukemia viruses. In addition, the S^+^L^−^ assay can detect RD114 feline retrovirus, feline leukemia virus types A, B and C, gibbon ape leukemia virus, and *Mus caroli* endogenous retrovirus (McERV) [16], [17], [21], thus the assays we used could have detected the presence of a broad range of gamma retroviruses.

To determine whether XMRV could persist in blood and to detect possible immune responses against XMRV, we assayed the ability of blood plasma to neutralize XMRV infectivity. We detected only minimal neutralization even under the most stringent condition of incubating a small amount of XMRV with undiluted, non-heat-inactivated plasma. At most, 75% of the XMRV was neutralized after incubation with plasma, suggesting that XMRV shed into the blood would have a long enough half-life to allow detection. Indeed, 7 of 21 plasma samples assayed in this way showed ≤10% neutralization (Fig. 1), indicating that human plasma has little innate neutralizing activity against XMRV. This result is consistent with a previous study of XMRV neutralization by sera from CFS and normal subjects, which showed 0 to 80% XMRV neutralization by undiluted non-heat-inactivated sera, and no XMRV neutralization by heat-inactivated sera [4], but is inconsistent with several other studies that detected relatively high neutralization of XMRV by human plasma or serum, even in those testing negative for XMRV by other criteria [22]–[24]. For example, Groom et al. [22] found examples of >50% neutralization of virus bearing XMRV Env proteins by heat-inactivated serum at 1∶40 and 1∶80 dilutions, and most of these positive results were from control subjects without prostate cancer or CFS. However, most of the positive sera also neutralized viruses bearing other Env proteins, including that of vesicular stomatitis virus, showing the neutralizing activity was generally nonspecific. Zhou et al. [24] found ∼30% neutralization of virus bearing XMRV Env by a 1∶80 dilution of heat-inactivated serum, with three sera showing ∼50% neutralization. All other assays performed indicated that all of these subjects were uninfected by XMRV.

Several factors may explain the differences in neutralizing antibody activities: i) heparin present in serum or plasma made from blood collected in heparinized tubes can nonspecifically inhibit virus infectivity, ii) repeated freezing and thawing of samples can inactivate complement resulting in reduced neutralization, iii) specific viral components of the virus used for neutralization studies can affect the results (for example, use of Gag proteins from HIV or other murine retroviruses in combination with XMRV Env [22]–[24]), and iv) cellular factors incorporated into virions during production of the virus used for neutralization can affect the result [25]–[27]. In our study and the study by Knox et al. [4], authentic XMRV produced from the human prostate cancer cell line 22Rv1 was used in the neutralization assays, while the studies of Groom et al. [22] and Zhou et al. [24] utilized viruses made with Moloney Gag-Pol proteins and XMRV Env, and were produced by transfection of human 293T cells. Additional antigens present in the latter viruses could account for some of the nonspecific neutralization observed.

In summary, we did not detect replication-competent XMRV in the plasma or EPS fluid from prostate cancer patients, nor did we detect significant levels of neutralizing antibodies in plasma. These data support the conclusion from other studies that XMRV has not entered the human population.

## Materials and Methods

### Human Subjects

Blood plasma and expressed prostatic fluid (EPS) samples used in the current study were obtained at the Cleveland Clinic following approval by the Cleveland Clinic Foundation Institutional Review Board. All samples were obtained from subjects with prostate cancer after written informed consent was obtained. Plasma samples were prepared from blood collected in standard EDTA tubes, EPS fluid was obtained by massage of excised prostate glands after prostatectomy, and all samples were stored at −70°C. Many of the plasma samples were frozen and thawed once for analysis, while others were frozen and thawed a limited number of times (Table 2).

### Cell Culture

*M. dunni* tail fibroblasts (dunni cells) [28], 293 human embryonic kidney cells [29], 22Rv1 prostate carcinoma cells (ATCC CRL-2505), HTX cells (an approximately diploid subclone of HT-1080 human fibrosarcoma cells) [5] and DU145 prostate cancer cells [30] were grown in Dulbecco’s modified Eagle’s medium with 4.5 g/l glucose and 10% fetal bovine serum (FBS). PG-4 feline cells [16] were grown in McCoy’s medium with 15% FBS. XMRV virus used in this study was harvested from 22Rv1 cells obtained directly from the ATCC.

### S^+^L^−^ and XMRV Neutralization Assays

PG-4 cells were seeded at 2.5×10^5^ per 6-cm dish on day 1. On day 2, plasma and EPS samples were thawed and portions of each sample (or culture medium as a no plasma control) were incubated with a small volume of infectious XMRV (harvested from 22Rv1 cells) for 15 to 30 minutes at room temperature, while the other portions of the plasma and EPS samples were kept on ice. Virus-spiked and untreated samples were added to the PG-4 cells in the presence of 4 µg/ml Polybrene. Cells were fed on day 3, and foci were counted on day 4 or 5. In some experiments the plasma was heat-inactivated at 56°C for 30 min before assay for virus neutralization.

### Marker Rescue Assay

Dunni and DU145 cells containing the LAPSN retroviral vector (dunni/LAPSN and DU145/LAPSN cells) were generated by exposing cells to helper-free LAPSN vector generated from PA317 retrovirus packaging cells [31] and then selecting the cells in G418 for 1 week to ensure the presence of the vector in all cells in the populations. The marker rescue assay was performed as follows. Dunni/LAPSN or DU145/LAPSN cells were seeded at 5×10^5^ per 6-cm dish on day 1 and were exposed to test samples (blood plasma or EPS) in the presence of 4 µg/ml Polybrene on day 2. The cells were then passaged for a month at high density (to facilitate virus spread) by trypsinizing and reseeding the cells at a 1∶10 dilution every time the cells became confluent. LAPSN production was then measured by feeding confluent cells, harvesting the medium the next day, removing cells by filtration (0.2 µm-pore-size surfactant-free cellulose acetate filters) or by centrifugation (4,000×g for 15 min), by adding medium samples with 4 µg/ml Polybrene to dunni or 293 cells seeded the day before at 5×10^4^ per well of 12-well plates, and by staining the cells for AP two days later.

### Virus Interference Assay

Replicating virus detected in the marker rescue assay was subjected to interference analysis using dunni cells chronically infected with either XMRV from 22Rv1 cells or with the *M. dunni* endogenous retrovirus (MDEV) from dunni cells. On day 1 the infected and uninfected dunni cells were seeded in 12-well dishes at 5×10^4^ per well. On day 2, the medium was replaced with 1 ml of medium containing 4 µg of Polybrene and 0.1 ml of medium harvested from the marker rescue assay cells. On day 4, the cells were stained for AP. The MDEV-infected dunni cells are resistant to MDEV but permissive to XMRV while the XMRV-infected dunni cells are resistant to XMRV but permissive to MDEV.

## References

[1] Urisman A, Molinaro RJ, Fischer N, Plummer SJ, Casey G (2006). Identification of a novel gammaretrovirus in prostate tumors of patients homozygous for R462Q RNASEL variant.. PLoS Path.

[2] Lombardi VC, Ruscetti FW, Das Gupta J, Pfost MA, Hagen KS (2009). Detection of an infectious retrovirus, XMRV, in blood cells of patients with chronic fatigue syndrome.. Science.

[3] Aloia AL, Sfanos KS, Isaacs WB, Zheng Q, Maldarelli F (2010). XMRV: A new virus in prostate cancer?. Cancer Res.

[4] Knox K, Carrigan D, Simmons G, Teque F, Zhou Y (2011). No evidence of murine-like gammaretroviruses in CFS patients previously identified as XMRV-infected.. Science.

[5] Knouf EC, Metzger MJ, Mitchell PS, Arroyo JD, Chevillet JR (2009). Multiple integrated copies and high-level production of the human retrovirus XMRV (xenotropic murine leukemia virus-related virus) from 22Rv1 prostate carcinoma cells.. J Virol.

[6] Paprotka T, Venkatachari NJ, Chaipan C, Burdick R, Delviks-Frankenberry KA (2010). Inhibition of xenotropic murine leukemia virus-related virus by APOBEC3 proteins and antiviral drugs.. J Virol.

[7] Smith RA, Gottlieb GS, Miller AD (2010). Susceptibility of the human retrovirus XMRV to antiretroviral inhibitors.. Retrovirology.

[8] Paprotka T, Delviks-Frankenberry KA, Cingöz O, Martinez A, Kung HJ (2011). Recombinant origin of the retrovirus XMRV.. Science.

[9] Hue S, Gray ER, Gall A, Katzourakis A, Tan CP (2010). Disease-associated XMRV sequences are consistent with laboratory contamination.. Retrovirology.

[10] Alberts B (2011). Retraction.. Science.

[11] Simmons G, Glynn SA, Komaroff AL, Mikovits JA, Tobler LH (2011). Failure to confirm XMRV/MLVs in the blood of patients with chronic fatigue syndrome: a multi-laboratory study.. Science.

[12] Dong B, Kim S, Hong S, Das Gupta J, Malathi K (2007). An infectious retrovirus susceptible to an IFN antiviral pathway from human prostate tumors.. Proc Natl Acad Sci USA.

[13] Hong S, Klein EA, Das Gupta J, Hanke K, Weight CJ (2009). Fibrils of prostatic acid phosphatase fragments boost infections with XMRV (xenotropic murine leukemia virus-related virus), a human retrovirus associated with prostate cancer.. J Virol.

[14] Ruscetti F, Lombardi V, Pfost M, Hagen K, Mikovits J (2010). http://oham.cancer.gov/objects/pdf/2010ICMAOI_Program_Book.pdf.

[15] Barton M (2011). http://rave.ohiolink.edu/etdc/view?acc_num=kent1309619878.

[16] Haapala DK, Robey WG, Oroszlan SD, Tsai WP (1985). Isolation from cats of an endogenous type C virus with a novel envelope glycoprotein.. J Virol.

[17] Miller AD, Wolgamot G (1997). Murine retroviruses use at least six different receptors for entry into *Mus dunni* cells.. J Virol.

[18] Lo SC, Pripuzova N, Li B, Komaroff AL, Hung GC (2010). Detection of MLV-related virus gene sequences in blood of patients with chronic fatigue syndrome and healthy blood donors.. Proc Natl Acad Sci USA.

[19] Miller AD, Bonham L, Alfano J, Kiem HP, Reynolds T (1996). A novel murine retrovirus identified during testing for helper virus in human gene transfer trials.. J Virol.

[20] Bonham L, Wolgamot G, Miller AD (1997). Molecular cloning of *Mus dunni* endogenous virus: an unusual retrovirus in a new murine viral interference group with a wide host range.. J Virol.

[21] Miller AD, Bergholz U, Ziegler M, Stocking C (2008). Identification of the myelin protein plasmolipin as the cell entry receptor for *Mus caroli* endogenous retrovirus.. J Virol.

[22] Groom HCT, Boucherit VC, Makinson K, Randal E, Baptista S (2010). Absence of xenotropic murine leukaemia virus-related virus in UK patients with chronic fatigue syndrome.. Retrovirology.

[23] Arnold RS, Makarova NV, Osunkoya AO, Suppiah S, Scott TA (2010). XMRV infection in patients with prostate cancer: novel serologic assay and correlation with PCR and FISH.. Urology.

[24] Zhou Y, Steffen I, Montalvo L, Lee TH, Zemel R (2012). Development and application of a high-throughput microneutralization assay: lack of xenotropic murine leukemia virus-related virus and/or murine leukemia virus detection in blood donors.. Transfusion.

[25] Rother RP, Fodor WL, Springhorn JP, Birks CW, Setter E (1995). A novel mechanism of retrovirus inactivation in human serum mediated by anti-α-galactosyl natural antibody.. J Exp Med.

[26] Takeuchi Y, Porter CD, Strahan KM, Preece AF, Gustafsson K (1996). Sensitization of cells and retroviruses to human serum by (α1–3) galactosyltransferase.. Nature.

[27] Takefman DM, Spear GT, Saifuddin M, Wilson CA (2002). Human CD59 incorporation into porcine endogenous retrovirus particles: implications for the use of transgenic pigs for xenotransplantation.. J Virol.

[28] Lander MR, Chattopadhyay SK (1984). A *Mus dunni* cell line that lacks sequences closely related to endogenous murine leukemia viruses and can be infected by ecotropic, amphotropic, xenotropic, and mink cell focus-forming viruses.. J Virol.

[29] Graham FL, Smiley J, Russell WC, Nairn R (1977). Characteristics of a human cell line transformed by DNA from human adenovirus type 5.. J Gen Virol.

[30] Mickey DD, Stone KR, Wunderli H, Mickey GH, Vollmer RT (1977). Heterotransplantation of a human prostatic adenocarcinoma cell line in nude mice.. Cancer Res.

[31] Miller AD, Buttimore C (1986). Redesign of retrovirus packaging cell lines to avoid recombination leading to helper virus production.. Mol Cell Biol.

